# Functional classification of secondary lymphedema

**DOI:** 10.1016/j.amsu.2019.10.025

**Published:** 2019-11-01

**Authors:** Jose Maria Pereira de Godoy, Maria de Fatima Guerreiro Godoy

**Affiliations:** a Cardiology and Cardiovascular Surgery Department of the Medicine School in São José Do Rio Preto (FAMERP), CNPq (National Council for Research and Development), Brazil; b Occupational Therapist Professor of the Post-Graduate Stricto Sensu in Medicine School in São José Do Rio Preto (FAMERP), Member of Research Group of the Clínica Godoy, Sao Jose Do Rio Preto, Brazil

**Keywords:** Lymphedema, Epidemiological classification, Secondary lymphedema

## Abstract

The epidemiological classifications of primary lymphedema based on age at emergence and the severity of the edema are fundamental to the transmission of information about this condition. In the secondary type, the lymphatic system is intact at birth but is injured during the course of life, causing a deficiency that leads to lymphedema. However, years of clinical experience and progress in treatment suggest new classifications to assist in the therapeutic planning of each patient. In clinical practice, we have observed inappropriate treatments that can cause more harm than good. Such observations are important and suggest the need for a proper diagnosis that considers all physiopathological processes involved for the establishment of the best form of treatment.

## 1 Letter to editor-perspective

Lymphedema is a specific type of edema caused by a failure in the formation or drainage of lymph. This condition may be congenital (primary) or acquired (secondary). In the primary type, damage to the lymphatic system is present since birth and may or may not lead to lymphedema during the course of one's life [1]. In the secondary type, the lymphatic system is intact at birth but is injured during the course of life, causing a deficiency that leads to lymphedema. The major causes of secondary lymphedema are cancer treatment that compromises the lymphatic system, particularly radiotherapy, trauma and infection, such as erysipelas and filariasis [[1], [2], [3]].

Lymphedema does not yet have a cure, but treatment can lead to the normalization or near normalization of the affected limb, including in cases of Stage III lymphedema, which is also known as elephantiasis [3]. The epidemiological classifications of primary lymphedema based on age at emergence and the severity of the edema are fundamental to the transmission of information about this condition. However, years of clinical experience and progress in treatment suggest new classifications to assist in the therapeutic planning of each patient.

The functional classification of secondary lymphedema is divided into two categories: the hypertensive and the non-hypertensive pattern. In this classification, we identify all physiopathological processes involved to assist in the therapeutic prognosis and the possibility of developing lymphedema.

With lymph node drainage, obstructions in proximal collectors can lead to a hypertensive pattern of the lymphatic vessels and the failure of these vessels over time. The pressure of lymphatic collectors is around 8 mmHg but can rise to around 100 mmHg when obstructed [4]. We denominate this physiopathological condition the hypertensive pattern, which is extremely important to the definition of treatment. In such cases, drainage can no longer be performed with sliding movements of the hands, as such movements can increase the pressure and injure the vessels. Thus, a different therapeutic option is needed – one that is adequate for this physiopathology.

This new classification is vital to the establishment of the most precise diagnosis and therapy suited to the physiopathology of the condition and the avoidance of inadequate therapies, such as those seen in current clinical practice. Sliding manual maneuvers over hypertensive collectors cannot be performed with the hypertensive pattern and alternative paths are suggested, such as cephalic and posterior chains in the upper limbs. However, we have no alternative collector in the trunk and another therapeutic option had been suggested by the authors for such cases [5].

This classification enables a better definition of the diagnosis, therapeutic strategy and prognosis. In clinical practice, a greater number of physiopathological processes involved increases the probability of developing lymphedema and increases the difficulty in both reducing the edema and maintaining the results. Surgery involving the removal of or injury to lymph nodes or lymphatic collectors constitutes a case of hypertensive lymphedema. When other physiopathological processes are added, such as radiotherapy, erysipelas, trauma or postoperative infection, inadequate therapies, such as massage with the application of force (contrary or not to the direction of the vessels) and inadequate exercises/activities, can lead to the aggravation of lymphedema. The idiopathic edema cycle has been detected in 10% of women with lymphedema and is an important aggravating factor that, if not treated, can render the treatment of the lymphedema unviable [6]. A previous study found that the addition of a physiopathological process increased the prevalence of lymphedema; with the addition of three or more physiopathological processes, nearly 100% of patients developed lymphedema following treatment for breast cancer [7].

In conclusion, a more adequate classification could assist in the establishment of adequate therapy for patients with lymphedema.

## Provenance and peer review

Not commissioned, externally peer reviewed.

## Ethical approval

This study no demand ethical approval because no involving patients.

## Sources of funding

N/A.

## Guarantor

Jose Maria Pereira de Godoy.

Maria de Fatima Guerreiro Godoy.

## Consent

N/A.

## Declaration of competing interest

No have conflict interest or financial support.
